# 4-Ethyl­anilinium perchlorate–18-crown-6 (1/1)

**DOI:** 10.1107/S1600536810031351

**Published:** 2010-08-11

**Authors:** De-Hong Wu

**Affiliations:** a College of Chemistry and Chemical Engineering, Southeast University, Nanjing 210096, People’s Republic of China

## Abstract

The asymmetric unit of the title compound, C_8_H_12_N^+^·ClO_4_^−.^C_12_H_24_O_6_, contains one half of the cationic [(C_2_H_5_—C_6_H_4_—NH_3_)(18-crown-6)]^+^ moiety and one half of the ClO_4_^−^ anion. Two O atoms of the crown ether, four C atoms and the N atom of the ethylanilinium unit and the Cl and two O atoms of the anion lie on a mirror plane. In the crystal structure, the –NH_3_^+^ group lies in the 18-crown-6 ring, forming a supra­molecular rotator–stator-like structure linked by intra­molecular N—H⋯O hydrogen bonds. The six O atoms of the crown ether lie approximately in a plane, the mean deviation being 0.1771 (3) Å; the N atom lies approximately 0.855 (3) Å from the centroid of the crown ether ring.

## Related literature

For background to 18-crown-6 compounds, see: Bajaj & Poonia (1988 ▶); Fender *et al.* (2002 ▶); Kryatova *et al.* (2004 ▶). For related structures. see: Dapporto *et al.* (1996 ▶); Pears *et al.* (1988 ▶).
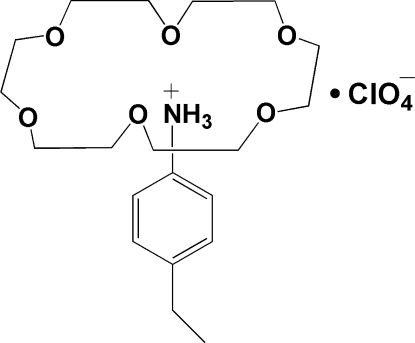


## Experimental

### 

#### Crystal data

C_8_H_12_N^+^·ClO_4_^−^·C_12_H_24_O_6_
*M* *_r_* = 485.95
Orthorhombic, 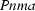

*a* = 16.6121 (13) Å
*b* = 11.4813 (13) Å
*c* = 12.8274 (16) Å
*V* = 2446.6 (5) Å^3^
*Z* = 4
Mo *K*α radiation
μ = 0.21 mm^−1^
*T* = 298 K
0.26 × 0.22 × 0.20 mm

#### Data collection

Rigaku Mercury2 diffractometer
Absorption correction: multi-scan (*CrystalClear*; Rigaku, 2005 ▶) *T*_min_ = 0.940, *T*_max_ = 0.960
21985 measured reflections
2525 independent reflections
1615 reflections with *I* > 2σ(*I*)
*R*_int_ = 0.086

#### Refinement

*R*[*F* ^2^ > 2σ(*F* ^2^)] = 0.064
*wR*(*F* ^2^) = 0.162
*S* = 1.05
2525 reflections
160 parameters
H-atom parameters constrained
Δρ_max_ = 0.32 e Å^−3^
Δρ_min_ = −0.29 e Å^−3^

### 

Data collection: *CrystalClear* (Rigaku, 2005 ▶); cell refinement: *CrystalClear*; data reduction: *CrystalClear*; program(s) used to solve structure: *SHELXS97* (Sheldrick, 2008 ▶); program(s) used to refine structure: *SHELXL97* (Sheldrick, 2008 ▶); molecular graphics: *SHELXTL* (Sheldrick, 2008 ▶); software used to prepare material for publication: *SHELXTL*.

## Supplementary Material

Crystal structure: contains datablocks I, global. DOI: 10.1107/S1600536810031351/jh2193sup1.cif

Structure factors: contains datablocks I. DOI: 10.1107/S1600536810031351/jh2193Isup2.hkl

Additional supplementary materials:  crystallographic information; 3D view; checkCIF report

## Figures and Tables

**Table 1:** Hydrogen-bond geometry (Å, °)

*D*—H⋯*A*	*D*—H	H⋯*A*	*D*⋯*A*	*D*—H⋯*A*
N1—H1*A*⋯O2^i^	0.89	2.20	2.919 (3)	138
N1—H1*A*⋯O3^i^	0.89	2.22	2.972 (3)	142
N1—H1*B*⋯O1	0.89	2.17	2.881 (4)	136
N1—H1*B*⋯O2	0.89	2.17	2.919 (3)	141
N1—H1*C*⋯O4	0.89	2.19	2.930 (4)	140
N1—H1*C*⋯O3	0.89	2.23	2.972 (3)	140

Symmetry code: (i) 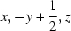.

**Fig. 1.**
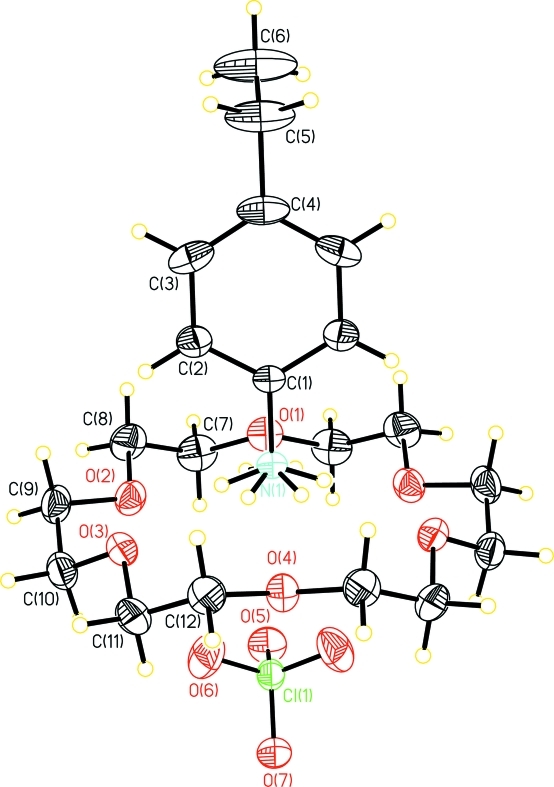
The molecular structure of the title compound, showing the atomic numbering scheme with 30% probability displacement ellipsoids.Unlabelled atoms are related to the labelled atoms by the (x, 1/2 - y, z) symmetry transformation.

**Fig. 2.**
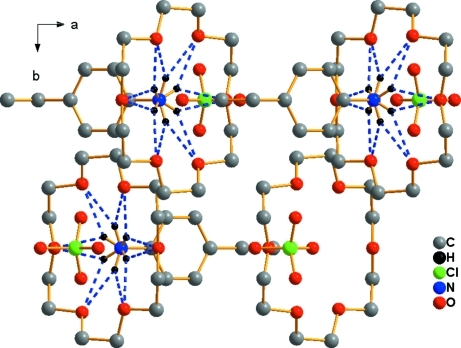
A view of the packing of the title compound, stacking along the c axis. Dashed blue lines indicate hydrogen bonds.

## supplementary crystallographic information

### Comment

Recently much attention has been devoted to crown ethers due to their ability to form non-covalent, H-bonding complexes with ammonium cations both in solid and in solution (Bajaj *et al.*, 1988; Fender *et al.*, 2002; Kryatova *et al.*, 2004). Both the nature of the ammonium cation (NH_4_^+^, RNH_3_^+^, *R*_2_NH_2_^+^, *etc*.) and the size of the crown ether can work on the stability and stoichiometry of these host–guest complexes. The host molecules combine with the guest species by intermolecular interaction, 18-Crown-6 has a high affinity for RNH_3_^+^cations and most studies of 18-crown-6 and its derivatives invariably showed a 1:1 stoichiometry with RNH_3_^+^ cations. For similar structures, see: Dapporto *et al.*, 1996; Pears *et al.*, 1988. In our laboratory, the title compound has been synthesized and its crystal structure is reported here.

The molecule of the title compound crystallizes in the orthorhombic *P*nma (No. 62) space group with an asymmetric unit consisting of one half cationic [(C_2_H_5_—C_6_H_4_—NH_3_)(18-crown-6)]^+^ moiety and one half isolated anionic ClO_4_^-^, In the crystal structure, The –NH_3_^+^ nests in the 18-crown-6 ring to form a supramolecular rotator-stator-like structure by intramolecular N—H···O hydrogen-bonded interactions between the –NH_3_^+^ and six oxygen atoms [O1, O2, O2A, O3, O3A and O4; symmetry code A of (*x*, 1/2 - *y*, *z*)] of the crown ether (Fig 1). Intermolecular N—H···O hydrogen distances fall within the normal range: 2.88–2.97Å (Table 2). The six oxygen atoms of the crown ether lie approximately in a plane with the mean deviation of 0.1771 (3) Å, the N1 atom aparts from the center (*Cg*1) of the crown ring about 0.855 (3)Å with the *Cg*2—N1—C6 angle of 176.3 (2)°. No formal hydrogen bonds are found between the ClO_4_^-^and –NH_3_^+^ moiety. the 10 non-hydrogen atoms (C1, C4, C5, C6, N1, O1, O4, O5, O7 and Cl1) located in a mirror plane with occupancy factor of 1/2, other atoms apart from the mirror plane can be produced by the (*x*, 1/2 - *y*, *z*) symmetry transformation. The –NH_3_^+^ moiety nests almost perpendicularly on the crown ring with an angle of 96.7°, the ethyl group is just in the mirror plane. The anionic ClO_4_^-^adopts a slightly distorted tetrahedral geometry with the Cl–O bond distances of 1.42–1.44Å and the F—B—F bond angles of 107.8–111.4°, no formal hydrogen bonds are found between the ClO_4_^-^and –NH_3_^+^ moiety.

### Experimental

The title compound, 4-ethylanilinium 18-crown-6 perchlorate was obtained as colorless block crystals by evaporation of themethanol solutioncontainingequal molar 18-crown-6 (*Aldrich*), perchloric acid (*Aldrich*)and 4-ethylbenzenamine (*Aldrich*)at room temperature. The dielectric constant of the compound as a function of temperature indicates that the permittivity is basically temperature-independent (ε = C/(T–T_0_)), suggesting that this compound is not ferroelectric or there may be no distinct phase transition occurring within the measured temperature range between 93 K and 434 K (below the compound melting point 474 K).

### Refinement

All H atoms were placed in calculated positions (N—H = 0.89 Å; C—H = 0.93Å for C*sp^2^* atoms and C—H = 0.96Å and 0.97Å for C*sp^3^* atoms), assigned fixed *U*_iso_ values [*U*_iso_ = 1.2*U*eq(C*sp^2^*) and 1.5*U*eq(C*sp^3^*, N)] and allowed to ride.

### Crystal data

**Table:**

C_8_H_12_N^+^·ClO_4_^−^·C_12_H_24_O_6_	*F*(000) = 1040
*M**_r_* = 485.95	*D*_x_ = 1.319 Mg m^−^^3^
Orthorhombic, *P**n**m**a*	Mo *K*α radiation, λ = 0.71073 Å
Hall symbol: -P 2ac 2n	Cell parameters from 16269 reflections
*a* = 16.6121 (13) Å	θ = 3.0–27.8°
*b* = 11.4813 (13) Å	µ = 0.21 mm^−^^1^
*c* = 12.8274 (16) Å	*T* = 298 K
*V* = 2446.6 (5) Å^3^	Block, colorless
*Z* = 4	0.26 × 0.22 × 0.20 mm

### Data collection

**Table:**

Rigaku Mercury2 diffractometer	2525 independent reflections
Radiation source: fine-focus sealed tube	1615 reflections with *I* > 2σ(*I*)
graphite	*R*_int_ = 0.086
Detector resolution: 13.6612 pixels mm^-1^	θ_max_ = 26.0°, θ_min_ = 3.0°
CCD_Profile_fitting scans	*h* = −20→20
Absorption correction: multi-scan (*CrystalClear*; Rigaku, 2005)	*k* = −14→14
*T*_min_ = 0.940, *T*_max_ = 0.960	*l* = −15→15
21985 measured reflections	

### Refinement

**Table:**

Refinement on *F*^2^	Primary atom site location: structure-invariant direct methods
Least-squares matrix: full	Secondary atom site location: difference Fourier map
*R*[*F*^2^ > 2σ(*F*^2^)] = 0.064	Hydrogen site location: inferred from neighbouring sites
*wR*(*F*^2^) = 0.162	H-atom parameters constrained
*S* = 1.05	*w* = 1/[σ^2^(*F*_o_^2^) + (0.0551*P*)^2^ + 1.8906*P*] where *P* = (*F*_o_^2^ + 2*F*_c_^2^)/3
2525 reflections	(Δ/σ)_max_ < 0.001
160 parameters	Δρ_max_ = 0.32 e Å^−^^3^
0 restraints	Δρ_min_ = −0.29 e Å^−^^3^

### Special details

**Table:**

Geometry. All e.s.d.'s (except the e.s.d. in the dihedral angle between two l.s. planes) are estimated using the full covariance matrix. The cell e.s.d.'s are taken into account individually in the estimation of e.s.d.'s in distances, angles and torsion angles; correlations between e.s.d.'s in cell parameters are only used when they are defined by crystal symmetry. An approximate (isotropic) treatment of cell e.s.d.'s is used for estimating e.s.d.'s involving l.s. planes.
Refinement. Refinement of *F*^2^ against ALL reflections. The weighted *R*-factor *wR* and goodness of fit *S* are based on *F*^2^, conventional *R*-factors *R* are based on *F*, with *F* set to zero for negative *F*^2^. The threshold expression of *F*^2^ > σ(*F*^2^) is used only for calculating *R*-factors(gt) *etc*. and is not relevant to the choice of reflections for refinement. *R*-factors based on *F*^2^ are statistically about twice as large as those based on *F*, and *R*- factors based on ALL data will be even larger.

### Fractional atomic coordinates and isotropic or equivalent isotropic displacement parameters (Å^2^)

**Table:**

	*x*	*y*	*z*	*U*_iso_*/*U*_eq_	Occ. (<1)
C1	0.2764 (2)	0.2500	0.1934 (3)	0.0389 (9)	
C2	0.30998 (18)	0.1465 (3)	0.1632 (2)	0.0556 (8)	
H2	0.2874	0.0763	0.1846	0.067*	
C3	0.3779 (2)	0.1472 (4)	0.1007 (3)	0.0696 (11)	
H3	0.4006	0.0768	0.0803	0.084*	
C4	0.4129 (3)	0.2500	0.0678 (3)	0.0660 (15)	
C5	0.4875 (3)	0.2500	0.0007 (4)	0.104 (2)	
H5A	0.4848	0.1821	−0.0442	0.125*	0.50
H5B	0.4848	0.3179	−0.0442	0.125*	0.50
C6	0.5599 (3)	0.2500	0.0462 (5)	0.148 (4)	
H6A	0.6010	0.2500	−0.0063	0.222*	
H6B	0.5653	0.1817	0.0889	0.222*	0.50
H6C	0.5653	0.3183	0.0889	0.222*	0.50
C7	0.2913 (2)	0.1464 (3)	0.5173 (3)	0.0683 (10)	
H7A	0.2449	0.1469	0.5631	0.082*	
H7B	0.3394	0.1430	0.5600	0.082*	
C8	0.2879 (2)	0.0424 (3)	0.4478 (3)	0.0671 (10)	
H8A	0.3319	0.0447	0.3981	0.080*	
H8B	0.2927	−0.0283	0.4887	0.080*	
C9	0.1956 (2)	−0.0628 (3)	0.3407 (3)	0.0619 (9)	
H9A	0.1970	−0.1274	0.3894	0.074*	
H9B	0.2357	−0.0765	0.2872	0.074*	
C10	0.1145 (2)	−0.0542 (3)	0.2926 (3)	0.0597 (9)	
H10A	0.0998	−0.1286	0.2621	0.072*	
H10B	0.0750	−0.0351	0.3456	0.072*	
C11	0.03706 (19)	0.0466 (3)	0.1679 (3)	0.0584 (9)	
H11A	−0.0029	0.0593	0.2218	0.070*	
H11B	0.0228	−0.0238	0.1303	0.070*	
C12	0.0379 (2)	0.1467 (3)	0.0954 (2)	0.0576 (9)	
H12A	0.0808	0.1373	0.0448	0.069*	
H12B	−0.0128	0.1510	0.0581	0.069*	
Cl1	0.09929 (7)	0.2500	0.78473 (9)	0.0546 (3)	
N1	0.20552 (17)	0.2500	0.2613 (2)	0.0391 (8)	
H1A	0.1908	0.3231	0.2745	0.059*	0.50
H1B	0.2174	0.2142	0.3209	0.059*	0.50
H1C	0.1653	0.2127	0.2298	0.059*	0.50
O1	0.29215 (17)	0.2500	0.4559 (2)	0.0555 (8)	
O2	0.21277 (13)	0.04384 (18)	0.39416 (17)	0.0562 (6)	
O3	0.11466 (12)	0.03337 (17)	0.21407 (16)	0.0499 (5)	
O4	0.05004 (17)	0.2500	0.1534 (2)	0.0499 (8)	
O5	0.1521 (2)	0.2500	0.8728 (3)	0.0782 (11)	
O6	0.11189 (18)	0.1476 (2)	0.7244 (2)	0.0994 (10)	
O7	0.0176 (2)	0.2500	0.8233 (3)	0.0882 (12)	

### Atomic displacement parameters (Å^2^)

**Table:**

	*U*^11^	*U*^22^	*U*^33^	*U*^12^	*U*^13^	*U*^23^
C1	0.034 (2)	0.048 (2)	0.035 (2)	0.000	0.0011 (16)	0.000
C2	0.0541 (19)	0.058 (2)	0.0549 (18)	−0.0007 (16)	0.0117 (15)	−0.0028 (17)
C3	0.053 (2)	0.094 (3)	0.061 (2)	0.017 (2)	0.0112 (17)	−0.016 (2)
C4	0.036 (2)	0.125 (5)	0.037 (2)	0.000	0.000 (2)	0.000
C5	0.048 (3)	0.215 (8)	0.049 (3)	0.000	0.008 (3)	0.000
C6	0.045 (3)	0.317 (13)	0.082 (4)	0.000	0.006 (3)	0.000
C7	0.064 (2)	0.089 (3)	0.0517 (19)	0.009 (2)	−0.0140 (17)	0.012 (2)
C8	0.061 (2)	0.070 (2)	0.071 (2)	0.0155 (19)	−0.0127 (18)	0.015 (2)
C9	0.076 (2)	0.0414 (19)	0.068 (2)	0.0107 (17)	0.0072 (19)	0.0112 (17)
C10	0.072 (2)	0.0426 (18)	0.065 (2)	−0.0073 (16)	0.0077 (18)	0.0025 (16)
C11	0.0546 (19)	0.054 (2)	0.067 (2)	−0.0138 (16)	−0.0057 (17)	−0.0073 (17)
C12	0.0550 (19)	0.059 (2)	0.0584 (19)	−0.0027 (17)	−0.0092 (16)	−0.0118 (18)
Cl1	0.0523 (6)	0.0445 (6)	0.0670 (7)	0.000	−0.0106 (6)	0.000
N1	0.0335 (16)	0.0436 (19)	0.0401 (18)	0.000	0.0010 (14)	0.000
O1	0.0567 (19)	0.068 (2)	0.0423 (17)	0.000	−0.0083 (14)	0.000
O2	0.0560 (13)	0.0481 (13)	0.0645 (13)	0.0096 (11)	−0.0071 (11)	0.0050 (11)
O3	0.0503 (12)	0.0424 (12)	0.0569 (12)	−0.0055 (9)	0.0017 (10)	0.0039 (10)
O4	0.0560 (18)	0.0449 (18)	0.0489 (17)	0.000	−0.0113 (14)	0.000
O5	0.073 (2)	0.092 (3)	0.070 (2)	0.000	−0.0167 (19)	0.000
O6	0.114 (2)	0.079 (2)	0.104 (2)	0.0202 (17)	−0.0095 (18)	−0.0371 (17)
O7	0.050 (2)	0.070 (3)	0.144 (4)	0.000	−0.003 (2)	0.000

### Geometric parameters (Å, °)

**Table:**

C1—C2	1.368 (4)	C9—C10	1.485 (4)
C1—C2^i^	1.368 (4)	C9—H9A	0.9700
C1—N1	1.465 (5)	C9—H9B	0.9700
C2—C3	1.385 (4)	C10—O3	1.423 (4)
C2—H2	0.9300	C10—H10A	0.9700
C3—C4	1.381 (4)	C10—H10B	0.9700
C3—H3	0.9300	C11—O3	1.427 (4)
C4—C3^i^	1.381 (4)	C11—C12	1.479 (4)
C4—C5	1.508 (6)	C11—H11A	0.9700
C5—C6	1.337 (7)	C11—H11B	0.9700
C5—H5A	0.9700	C12—O4	1.415 (3)
C5—H5B	0.9700	C12—H12A	0.9700
C6—H6A	0.9600	C12—H12B	0.9700
C6—H6B	0.9600	Cl1—O6	1.423 (3)
C6—H6C	0.9600	Cl1—O6^i^	1.423 (3)
C7—O1	1.427 (4)	Cl1—O5	1.430 (3)
C7—C8	1.491 (5)	Cl1—O7	1.444 (4)
C7—H7A	0.9700	N1—H1A	0.8900
C7—H7B	0.9700	N1—H1B	0.8900
C8—O2	1.425 (4)	N1—H1C	0.8900
C8—H8A	0.9700	O1—C7^i^	1.427 (4)
C8—H8B	0.9700	O4—C12^i^	1.415 (3)
C9—O2	1.432 (4)		
			
C2—C1—C2^i^	120.5 (4)	C10—C9—H9A	109.9
C2—C1—N1	119.72 (19)	O2—C9—H9B	109.9
C2^i^—C1—N1	119.72 (19)	C10—C9—H9B	109.9
C1—C2—C3	119.4 (3)	H9A—C9—H9B	108.3
C1—C2—H2	120.3	O3—C10—C9	109.8 (3)
C3—C2—H2	120.3	O3—C10—H10A	109.7
C4—C3—C2	121.7 (4)	C9—C10—H10A	109.7
C4—C3—H3	119.2	O3—C10—H10B	109.7
C2—C3—H3	119.2	C9—C10—H10B	109.7
C3^i^—C4—C3	117.3 (4)	H10A—C10—H10B	108.2
C3^i^—C4—C5	121.3 (2)	O3—C11—C12	109.6 (3)
C3—C4—C5	121.3 (2)	O3—C11—H11A	109.8
C6—C5—C4	119.3 (5)	C12—C11—H11A	109.8
C6—C5—H5A	107.5	O3—C11—H11B	109.8
C4—C5—H5A	107.5	C12—C11—H11B	109.8
C6—C5—H5B	107.5	H11A—C11—H11B	108.2
C4—C5—H5B	107.5	O4—C12—C11	108.8 (2)
H5A—C5—H5B	107.0	O4—C12—H12A	109.9
C5—C6—H6A	109.5	C11—C12—H12A	109.9
C5—C6—H6B	109.5	O4—C12—H12B	109.9
H6A—C6—H6B	109.5	C11—C12—H12B	109.9
C5—C6—H6C	109.5	H12A—C12—H12B	108.3
H6A—C6—H6C	109.5	O6—Cl1—O6^i^	111.4 (3)
H6B—C6—H6C	109.5	O6—Cl1—O5	109.85 (15)
O1—C7—C8	109.7 (2)	O6^i^—Cl1—O5	109.85 (15)
O1—C7—H7A	109.7	O6—Cl1—O7	108.94 (16)
C8—C7—H7A	109.7	O6^i^—Cl1—O7	108.94 (16)
O1—C7—H7B	109.7	O5—Cl1—O7	107.8 (2)
C8—C7—H7B	109.7	C1—N1—H1A	109.5
H7A—C7—H7B	108.2	C1—N1—H1B	109.5
O2—C8—C7	108.2 (3)	H1A—N1—H1B	109.5
O2—C8—H8A	110.1	C1—N1—H1C	109.5
C7—C8—H8A	110.1	H1A—N1—H1C	109.5
O2—C8—H8B	110.1	H1B—N1—H1C	109.5
C7—C8—H8B	110.1	C7^i^—O1—C7	113.0 (3)
H8A—C8—H8B	108.4	C8—O2—C9	113.3 (2)
O2—C9—C10	108.8 (3)	C10—O3—C11	111.6 (2)
O2—C9—H9A	109.9	C12—O4—C12^i^	113.9 (3)

Symmetry codes: (i) *x*, −*y*+1/2, *z*.

### Hydrogen-bond geometry (Å, °)

**Table:**

*D*—H···*A*	*D*—H	H···*A*	*D*···*A*	*D*—H···*A*
N1—H1A···O2^i^	0.89	2.20	2.919 (3)	138.
N1—H1A···O3^i^	0.89	2.22	2.972 (3)	142.
N1—H1B···O1	0.89	2.17	2.881 (4)	136.
N1—H1B···O2	0.89	2.17	2.919 (3)	141.
N1—H1C···O4	0.89	2.19	2.930 (4)	140.
N1—H1C···O3	0.89	2.23	2.972 (3)	140.

Symmetry codes: (i) *x*, −*y*+1/2, *z*.
